# Remimazolam Pilot for Office-Based Dental Sedation: Adverse Events, Awareness and Outcomes

**DOI:** 10.3390/jcm12237308

**Published:** 2023-11-25

**Authors:** Rudi Swart, Sabine S. A. Maes, David Cavanaugh, Keira P. Mason

**Affiliations:** 1 Advanced Sedation Ltd., London CM4 0EG, UK; dr.r.swart@gmail.com; 2 Department of Anesthesia, University Hospital Antwerp, 2650 Edegem, Belgium; sabine.maes@uza.be; 3 Boston Biostatistical Consulting, North Reading, MA 01864, USA; dmcav6@gmail.com; 4 Department of Anesthesia, Critical Care and Pain Medicine, Boston Children’s Hospital, Boston, MA 02115, USA

**Keywords:** remimazolam, alfentanil, sedation, dental sedation, office-based sedation, safety, intravenous sedation, adverse events

## Abstract

In recent years, remimazolam has gained approval for use in adult procedural sedation in both the United Kingdom and the United States, potentially offering an alternative to conventional sedatives like propofol and midazolam for procedural sedation. However, there is a limited body of literature that systematically reviews the outcomes of a remimazolam-alfentanil combination protocol for routine office-based dental procedures. The primary objective of this pilot study was to assess the occurrence of significant adverse events associated with the use of a remimazolam-alfentanil sedation protocol for adult dental procedures. Secondary outcomes included evaluating physiological responses, sedation effectiveness, patient and clinician satisfaction and the incidence of intraprocedural awareness. Notably, no significant adverse events were reported among the 25 adult subjects who received remimazolam and alfentanil, and all dental procedures were successfully completed. Patients and clinicians expressed high levels of satisfaction, and patients did not report any distressing memories associated with the dental procedure. These findings suggest that in a limited cohort, the remimazolam-alfentanil regimen appears to be well tolerated and effective for office-based dental procedures in adult patients, with a low risk of adverse events, acceptable hemodynamic effects, rapid onset and recovery and minimal intraoperative awareness. This study provides valuable insights into the potential use of the remimazolam-alfentanil combination in dental sedation practice.

## 1. Introduction

Remimazolam is a novel sedative agent. It is classified as an intravenous benzodiazepine and is a selective ligand for GABA-A receptors in the central nervous system. As a modification of midazolam, remimazolam is metabolized by tissue esterases to inactive metabolites. With a more rapid elimination half-life than midazolam, remimazolam may offer the advantage of faster induction to targeted sedation depth and more rapid recovery.

To date, worldwide, remimazolam has limited adult labelling in only a few countries in very recent years [1]. Remimazolam sedation has been described for adult gastrointestinal endoscopies with results that suggest a similar induction and better recovery profile to propofol [2,3,4,5,6]. When compared to midazolam, remimazolam offers the benefits of faster recovery and a constant elimination half-life during prolonged exposure. The sedative effects of remimazolam can be antagonized by flumazenil [1]. Alfentanil is a synthetic opioid analgesic, and compared to fentanyl and sufentanil, it has a more rapid analgesic onset and time-to-peak effect, as well as the shortest distribution and elimination half-life [7].

The combination of remimazolam and alfentanil has been described for gastrointestinal endoscopies as well as for some simple dental procedures (third molar extractions) [8,9]. There is limited experience with remimazolam for office-based dental procedures [10]. Although remimazolam use has been reported for select maxillofacial surgical procedures, to date, there is no published literature that reviews the outcomes of a protocol that uses a remimazolam-alfentanil combination for a wide range of routine office-based dental procedures [10,11].

Our primary objective in this pilot study was to determine whether there are any significant adverse events related to a remimazolam-alfentanil sedation protocol for office-based adult dental procedures. Adverse events were identified using a standardized objective template created by the International Committee for the Advancement of Procedural Sedation [12]. Our secondary outcome was to evaluate the physiological response, sedation profile, patient and clinician satisfaction and presence of intraprocedural awareness associated with our remimazolam-alfentanil combination.

## 2. Materials and Methods

Between 7 October 2022 and 19 April 2023, 146 adult patients scheduled for office-based dental procedures were offered the option of receiving single or combination sedation with midazolam, propofol, ketamine, alfentanil or a combination of alfentanil and remimazolam.

The remimazolam combination was offered to the patients as a recently approved sedative with the reported possibility of an improved recovery profile. There was an additional cost associated with choosing for this type of sedation. Overall, 25 patients requested the remimazolam-alfentanil protocol. All patients signed informed consent for the sedation and consented to have the collected data used without Health Insurance Portability and Accountability Act (HIPAA) Identifiers for quality improvement activity, research and publication. Per the United States Department of Health and Human Services definition and National Health Service Medical Research Council of the United Kingdom (UK), Institutional Review Board (IRB) approval was not required for this quality assurance initiative intended to improve the quality of patient care [13,14]. Remimazolam was administered following approved guidelines for remimazolam sedation and alfentanil was delivered as an adjunct per a protocol that the provider (RS) has established as his standard of care [15]. Physiologic data, patient and clinician satisfaction and patient awareness metrics were collected on all patients as part of routine sedation documentation and the ongoing quality assurance initiative. All sedation was administered by a single provider (RS).

The sample size was calculated to evaluate the number of adverse events reported based on a 20% prevalence of experiencing an adverse event of any kind (sentinel to minor) vs. a 1% prevalence (incidence of sentinel events).

The number of subjects plus allowing for an additional 20% required a total of 25 subjects.

Inclusion criteria were adult patients ≥ 18 years old, ASA 1 and 2, with no history of Obstructive Sleep Apnea, no benzodiazepine allergies, Body Mass Index < 40 kg/m^2^ and no uncontrolled gastroesophageal reflux or vomiting.

All patients were nil per os (NPO) for solids and clear fluids for a minimum of four hours. All patients were pre-screened using a template-designed preoperative questionnaire to ensure a comprehensive medical history, the presence of any allergies and a review of the organ systems. Sedation monitoring and documentation followed the American Society of Anesthesiologists (ASA) Moderate Sedation Guidelines and the United Kingdom IACSD Guidelines [15,16]. Standard-of-care monitoring equipment included non-invasive blood pressure (Withings BPM Connect Blood Pressure), oxygen saturation (Nellcor Bedside SpO_2_ Patient Monitoring System PM100N) and a waveform carbon dioxide monitor (Newtech NT1D Handheld Mainstream EtCO_2_). Baseline vital signs were recorded prior to sedation initiation and during the sedation until discharge criteria were met. The protocol followed the approved UK guidelines for the use of remimazolam combined with an opioid, which specify the administration of an initial remimazolam bolus of 2.5 mg for frail patients (ASA 3–4, or patients < 50 kg) or a bolus of 5 mg for ASA 1 and 2 patients under 65 years of age over 1 min followed by alfentanil 100–200 micrograms as clinically warranted. Supplemental boluses of remimazolam of 1.25–2.5 mg were administered at least 2 min after the initial bolus under continuous assessment of the level of sedation [17,18].

Vital signs were monitored and recorded until discharge criteria were met. Sedation depth was assessed using the modified Ramsay Sedation Scale (RSS) every 10 min from the initiation of sedation until discharge home [19]. Dental surgery commenced once the desired sedation depth of minimum RSS 3 was achieved, and the duration of surgery was documented, starting from the initial injection of local anesthesia. Alfentanil (100–200-microgram bolus) was administered when appropriate and alternated with remimazolam (2.5-milligram bolus) to maintain the desired sedation level. Administration of the sedative and opioid was stopped when the dental procedure was completed.

In recovery, after discharge criteria were met (minimum Aldrete score of 8), each patient completed a Patient Sedation Satisfaction Interview and a validated amnesia questionnaire to assess for awareness and dreaming during the sedation [20,21,22]. The dentist completed the Clinician Sedation Satisfaction Interview to evaluate the level of sedation and patient cooperation during the dental procedure [22].

Adverse events were collected and evaluated based on the International Committee for the Advancement of Procedural Sedation using the standardized metrics for procedural sedation outcome [12].

## 3. Results

Summaries of data were calculated using descriptive statistics: number of patients (*n*), mean, standard deviation (SD), median, minimum and maximum, as well as frequency counts with percentages for categorical variables. Baseline demographic and disease characteristic information: gender, age, height, weight and body mass index (BMI); ASA score; Ramsay low and Ramsay high were summarized with descriptive statistics. Procedural and drug dosage amount were also summarized.

Patient awareness questions, the Clinical Sedation Satisfaction Index (CSSI) and the Patient Sedation Satisfaction Index (PSSI) were summarized by the number of subjects with each response.

Vital signs (End Tidal CO_2_, Oxygen Saturation, Respiratory Rate, Heart Rate) were collected prior to procedure and every 10 min. Results at each timepoint, as well as the change from baseline and percent change from baseline, were calculated. Similarly, blood pressure was collected at various timepoints and was summarized in the same manner as the vital sign data.

Statistical analyses were performed using SAS^®^ statistical software (version 9.4, SAS^®^ Institute, Cary, NC, USA).

Baseline and demographics data were summarized using descriptive statistics and are shown in Table 1. There were 25 subjects in the study: 17 (68.0%) females and 8 (32.0%) males. The median age was 60 years old with a range from 21 to 82 years old.

There were no adverse events experienced by any subject. All subjects received remimazolam and alfentanil, and all procedures were successfully completed. The duration of procedure was a mean (SD) of 67.4 (42.82) minutes, with the duration of remimazolam administration a mean (SD) of 61.8 (44.01) minutes. The mean (SD) time to discharge after treatment was 35.4 (17.91) minutes. The median (interquartile range) time to discharge was 30 (25, 41.25) minutes. The mean (SD) time to discharge following the last dose of remimazolam was 41.4 (20.59) minutes. The median (interquartile range) time to discharge following the last dose of remimazolam was 40 (35, 45) minutes. Overall, 19 (76%) subjects had a Ramsay high score of 3 or 3a. These results are summarized in Table 1.

Table 2 summarizes the responses of five questions upon receiving the anesthesia. There were nine (36.0%) subjects who responded that the time before or after the initial dose, respectively, was the last thing they remembered before going to sleep. In total, 92% did not recall the injection of the local anesthesia, which marked the initiation of the surgical procedure. One patient recalled the start of the procedure. In total, 52% did not have any recall of the procedure, and 14 (56.0%) subjects said that the worst thing about the procedure was the pre-operative anxiety.

The percent change from baseline MAP for each patient’s lowest and highest recorded MAP was calculated. The number of patients in each group was measured by categorizing them into three groups: those with little change from baseline (from 0% to 20% change), those with a moderate change from baseline (from 20% to 30% change) and those with a large change from baseline (30% or greater).

**Figure 1:**
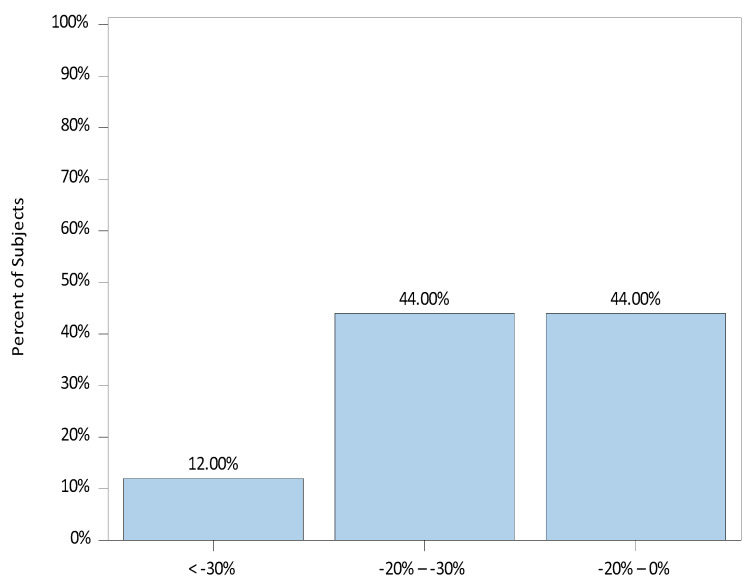
Percent Change from Baseline to Lowest Mean Arterial Blood Pressure (MAP).

The median (range) percent change from baseline to the lowest MAP was −23.3 (−36.9, 0). There were 23 patients (92.0%) with a drop in MAP below their baseline, and 2 (8.0%) showed no drop. The distribution of percent changes from baseline to the lowest MAP is illustrated in Figure 1.

**Figure 2:**
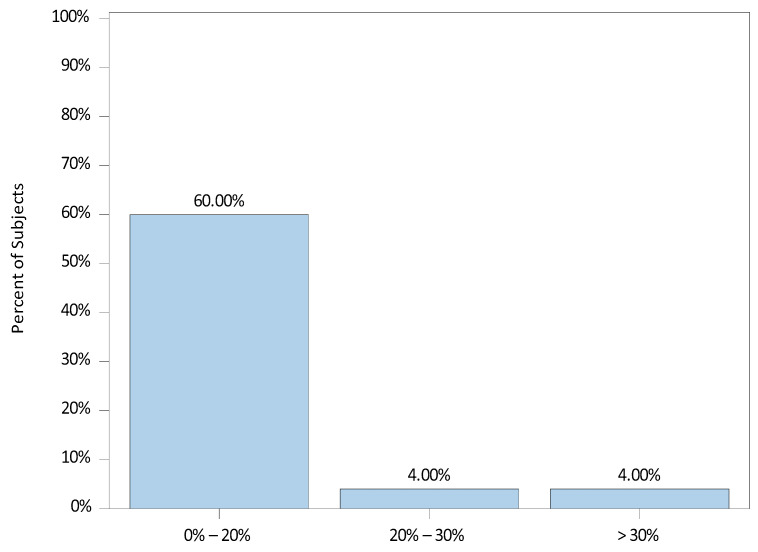
Percent Change from Baseline to Highest Mean Arterial Blood Pressure (MAP).

The median (range) percent change from baseline to the highest MAP was 0.0 (−11.1, 36.5). There were 9 (36.0%) patients with an increase in the highest MAP and 16 (64.0%) without an increase. The distribution of percent changes from baseline to the highest MAP is illustrated in Figure 2.

**Figure 3:**
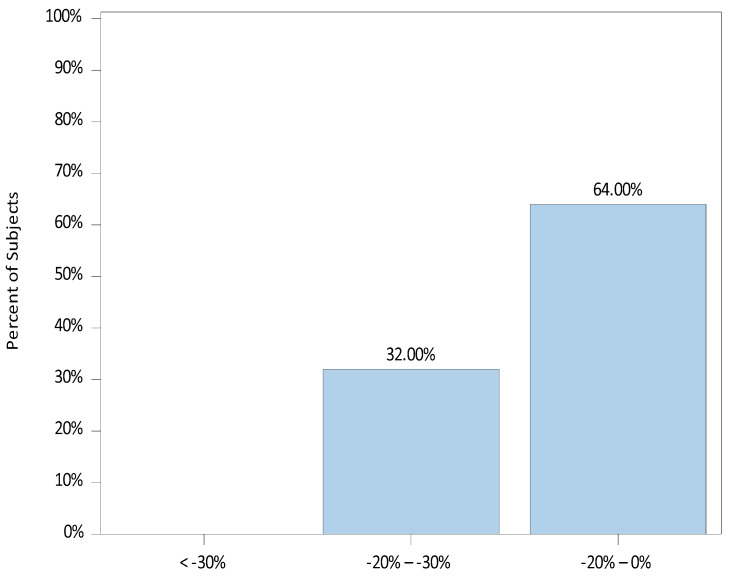
Percent Change from Baseline to Lowest Heart Rate (HR).

Similarly for heart rate (HR), the percent change from baseline HR for each patient’s lowest and highest recorded HR was calculated and grouped into the three categories of change. The median (range) percent change from baseline to lowest HR was −13.3 (−29.4, 3.2). Of the 25 patients, 22 (88.0%) experienced a decrease in HR below their baseline, whereas 3 (12.0%) showed no decrease. The distribution of percent changes from baseline to the lowest HR is illustrated in Figure 3.

**Figure 4:**
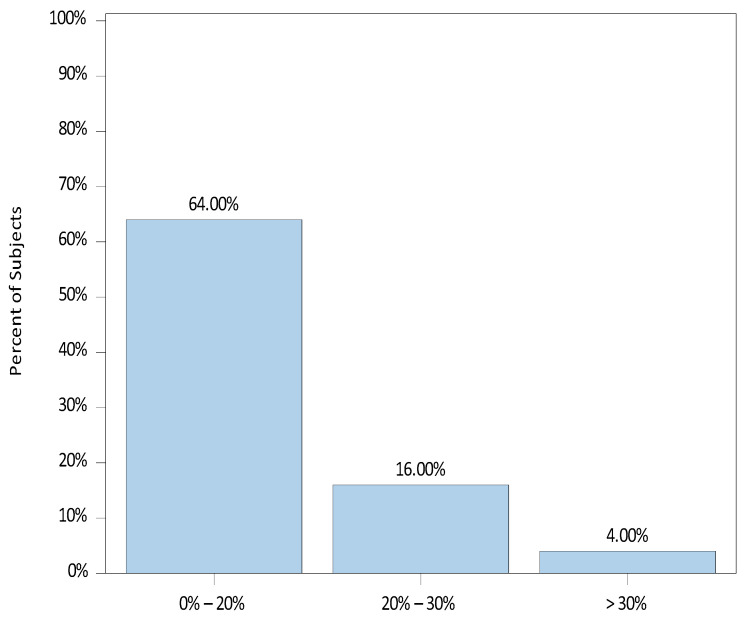
Percent Change from Baseline to Highest Heart Rate (HR).

The median (range) percentage difference from the baseline to the highest HR was recorded as 9.2 (−3.3, 34.2). Of the 25 patients, 17 (68.0%) experienced an increase in HR above their baseline, whereas 8 (32.0%) showed no increase. The distribution of percent changes from baseline to the highest HR is illustrated in Figure 4.

The Clinical Sedation Satisfaction Index (CSSI) is summarized by question and response in Table 3. Overall, 24 (100%) of subjects responded that they were very satisfied with the procedure and with the sedation part of the procedure. Moreover, 22 subjects (91.7%) responded they were satisfied with the effectiveness of the sedation received and the effect the sedation had on the procedure.

The Patient Sedation Satisfaction Index (PSSI) is summarized by question and response in Table 4. Overall, 24 (96%) of subjects responded that they were very satisfied with their overall satisfaction with the sedation experience. There were 23 (92.0%) of subjects who were very satisfied with the pain associated with the sedation delivery, the amount of sedation received (enough to make you drowsy or go to sleep), the amount of nausea after the procedure, and the ease of recovery after the procedure.

## 4. Discussion

Remimazolam is the newest sedative to be introduced since dexmedetomidine, over 20 years ago. The early phase 1 trials with remimazolam demonstrated that remimazolam can elicit a dose-dependent sedation that can achieve sedation depths similar to midazolam but with a faster recovery profile. Its maximum effect was shown to be at 3 min [23]. The pharmacokinetic profile of remimazolam is similar in adults and children with a high clearance and short context-sensitive half-life of 7 min vs. 17 min in adults and children, respectively [24,25]. The electroencephalogram beta ratio in response to remimazolam shows a 0.79 prediction probability score for the Modified Observer’s Assessment of Alertness and Sedation [26].

Propofol, a common agent used for ambulatory procedures, has been compared to remimazolam for upper gastrointestinal endoscopes in phase 3 trials performed in China. The time to achieve sedation and recovery were slightly faster with propofol (by approximately one minute), but remimazolam exhibited less hypotension, less respiratory depression and fewer adverse events [27]. In combination with alfentanil, propofol can exhibit more respiratory depression in spontaneously ventilating patients than that seen with the alfentanil-remimazolam combination. Hypotension was more common in those who received propofol [28]. A meta-analysis of randomized controlled trials comparing propofol to remimazolam for procedural sedation demonstrated less bradycardia, hypotension and respiratory depression with remimazolam but with a similar efficacy and side effect profile (nausea, vomiting, dizziness) [29]. The induction and recovery time comparing propofol and remimazolam (when reversed with flumazenil) for ambulatory procedures has been shown to be comparable [30]. When compared to propofol, remimazolam may lessen the degree of short-term cognitive dysfunction in the elderly (over 65 years) and is similar to dexmedetomidine in reducing post op cognitive dysfunction seen at 3 and 7 days [31,32].

There are some reports of remimazolam being used for oral surgery, maxilla-facial and dental procedures. When compared to midazolam, remimazolam has been shown to be more successful than midazolam in achieving adequate sedation depth to complete oral maxilla-facial surgical procedures [10]. For the removal of impacted wisdom teeth, remimazolam produced faster onset and recovery, fewer post-operative side effects and greater patient satisfaction than midazolam [11]. A small series that compared remimazolam-alfentanil to propofol-alfentanil for third molar extraction described faster recovery and discharge with the remimazolam group [9].

One patient in our study had a delayed discharge time (100 min vs. 41 min average) because of nausea and vomiting after becoming fully alert. In this particular patient, a large amount of blood had been swallowed during the procedure, probably due to inadequate suctioning. The prolonged recovery time in the situation did not reflect the sedation technique or delivery.

There are some adverse events that have been reported with remimazolam along with some precautionary recommendations. Remimazolam should be administered with normal saline, as lactate ringer can create precipitate which has occluded venous catheters [33,34]. Case reports of anaphylaxis have been recently reported. The etiology, hypothesized to be Ige mediated from remimazolam or non-IgE mediated from the dextran, has not yet been determined [35] Flumazenil, often used to reverse the sedative effects of remimazolam in order to expedite recovery, competed with remimazolam at the receptor. Re-sedation after flumazenil, particularly when flumazenil is administered in large dosages, is a risk [36].

Our pilot study validates prior studies which demonstrate the rapid induction and recovery profile of remimazolam. Our average time to achieve favorable conditions to begin the procedure averaged 5 min, with an average of 34 and 41 min to meet discharge criteria after the last dose of remimazolam and the completion of the procedure, respectively. We did not administer flumazenil to any patient as there was no clinical indication. Remimazolam seems to have anterograde amnesia, with the majority of our patients having no recall from the time of initial dosing until after completion of the procedure. In total, 100% of the patients were very satisfied with the sedation, and 88% of those who had received alternative sedatives for past procedures were very satisfied with this remimazolam-alfentanil combination. Overall, 96% reported being satisfied or very satisfied with their ability to think clearly after the procedure. Our initial results are encouraging, suggesting that remimazolam is efficacious, safe and with minimal effects on post-procedure cognition. Further studies will be needed to look specifically at each of these components on a larger scale, with patients over a range of ASA status and ages.

Our procedure had some limitations. Our patients were healthy, non-obese adults who were not at the extreme of old age. None of the patients had hepatic or renal dysfunction, either of which can affect the pharmacokinetics [37]. Our induction time, procedure time and time to meet discharge criteria was relatively short, lending itself to the office-based setting. We did not reverse any of the patients with flumazenil, a practice often utilized in order to expedite awakening and return of psychomotor function and memory retention [38,39]. Although we did not report any significant adverse events in this pilot study, it is possible that larger studies are needed to detect occurrences of sentinel, life threatening adverse events which occur at a low incidence. We do not know the incidence of significant adverse events with remimazolam in the adult population, as large studies and reports of significant adverse events (aside from anaphylaxis) have not yet been widely reported. Larger studies are needed to determine the profile of remimazolam on the extremes of age, those with organ dysfunction and those with longer procedures. The effect of flumazenil on psychomotor return as well as recovery should be evaluated. Although none of our patients seemed to suffer from procedural awareness, follow-up studies should be performed to follow those patients in terms of their recall. As a sedative with negligible respiratory effects and minimal effects on cognitive function, the value of remimazolam in ambulatory and outpatient surgical procedures may be far-reaching.

## 5. Conclusions

Remimazolam may offer a safe alternative to propofol and midazolam for office-based dental procedures in adult patients, with minimal risk of adverse events, clinically acceptable hemodynamic effects, rapid induction and emergence and negligible intraoperative awareness.

## Figures and Tables

**Table 1:** Baseline, demographics and procedural data.

Variable	N (%) or Mean (SD)	Min, Max
Gender		
Female	17 (68.0)	
Male	8 (32.0)	
Weight (kg)	76.9 (19.56)	38.0, 123.0
Age (years)	55.7 (18.48)	21.0, 82.0
Height (cm)	169.7 (8.80)	152.0, 182.0
BMI (kg/m^2^)	27.6 (6.49)	15.0, 40.0
ASA		
1	11 (44.0)	
2	14 (56.0)	
Adverse Events		
No adverse events	25 (100.0)	
Remimazolam total dose (mg)	30.9 (14.12)	12.5, 60.0
Remimazolam administered (mg/kg/duration in minutes)	0.0083557 (0.0045309)	0.0036134, 0.0263158
Remimazolam duration administered (min)	61.8 (44.01)	15.0, 205.0
Alfentanil total dose (microgram)	1276.0 (1104.26)	200.0, 4400.0
Alfentanyl total dose (microgram/kg)	16.3 (13.31)	2.4, 62.9
Duration of procedure (min)	67.4 (42.82)	15.0, 209.0
Intraprocedural Modified Ramsay sedation scale	3.2 (0.47)	2.0, 4.0
Aldrete Discharge Score	9.8 (0.52)	8.0, 10.0
Time to discharge after treatment (min)	35.4 (17.91)	15.0, 100.0
Discharge after last dose of remimazolam (min)	41.4 (20.59)	10.0, 120.0
Start of procedure after initial dose (min)	4.9 (2.35)	2.0, 10.0

**Table 2:** Awareness and dreaming questionnaire.

Question	Response *n* (%)	
What is the last thing you remember before going to sleep?	A. Time before initial dose administered	9 (36.0)
	B. Time initial dose delivered	5 (20.0)
	C. Time after initial dose before LA	9 (36.0)
	D. Time LA given	1 (4.0)
	F. Time procedure started	1 (4.0)
What is the first thing you remember waking up?	H. Immediately after the procedure	12 (48.0)
	I. More than 5 min after the procedure	13 (52.0)
Do you remember anything between going to sleep and waking up?	No discomfort only instructions/talking	9 (36.0)
	Nothing	13 (52.0)
	Some events unrelated to the procedure	3 (12.0)
Did you dream during the procedure?	Nil	2 (8.0)
	No	16 (64.0)
	Not sure	1 (4.0)
	Yes	1 (4.0)
	Yes, but can’t remember what	5 (20.0)
What was the worst thing about your operation/procedure?	Intravenous cannulation	4 (16.0)
	Post procedure numbness, teeth extraction, denture	3 (12.0)
	Paying the bill	1 (4.0)
	Post-operative vomiting	1 (4.0)
	Pre-operative anxiety	14 (56.0)
	Pre-operative fasting	1 (4.0)
	Procedure being delayed	1 (4.0)

**Table 3:** Clinician Sedation Satisfaction Index (CSSI).

Question	Very Satisfied	Satisfied	Somewhat Satisfied	Neither Satisfied or Dissatisfied	Somewhat Dissatisfied	Dissatisfied	Very Dissatisfied
Effectiveness of the sedation received	22 (91.7)	2 (8.3)	0	0	0	0	0
Effect the sedation had on the procedure	22 (91.7)	2 (8.3)	0	0	0	0	0
Patient’s ability to communicate post operatively	24 (100.0)	0	0	0	0	0	0
Patient’s ability to retain post operative information	19 (79.2)	4 (16.7)	0	1 (4.2)	0	0	0
Recovery time associated with the sedation	21 (87.5)	3 (12.5)	0	0	0	0	0
Your overall satisfaction with the procedure	24 (100.0)	0	0	0	0	0	0
Your overall satisfaction with the sedation part of the procedure	24 (100.0)	0	0	0	0	0	0
Patient’s cooperation level	23 (95.8)	0	1 (4.2)	0	0	0	0
Overall ease of the procedure	23 (95.8)	1 (4.2)	0	0	0	0	0
Method of sedation compared with other methods of sedation	21 (87.5)	3 (12.5)	0	0	0	0	0

Percentages based on the number of subjects that responded.

**Table 4:** Patient Sedation Satisfaction Index (PSSI).

Question	Very Satisfied	Satisfied	Somewhat Satisfied	Neither Satisfied or Dissatisfied	Somewhat Dissatisfied	Dissatisfied	Very Dissatisfied
Pain associated with the sedation delivery	23 (92.0)	2 (8.0)	0	0	0	0	0
Amount you remember during the procedure	19 (76.0)	3 (12.0)	2 (8.0)	1 (4.0)	0	0	0
Amount of drowsiness with the medication	17 (68.0)	4 (16.0)	2 (8.0)	1 (4.0)	0	1 (4.0)	0
Amount of sedation you received (enough to make you drowsy or go to sleep)	23 (92.0)	1 (4.0)	0	1 (4.0)	0	0	0
Amount of nausea after the procedure	23 (92.0)	1 (4.0)	0	0	0	0	1 (4.0)
Amount you remember about the procedure	20 (80.0)	3 (12.0)	1 (4.0)	0	1 (4.0)	0	0
Length of time you felt the effects of the sedation received	22 (88.0)	3 (12.0)	0	0	0	0	0
Drowsiness after the procedure	14 (56.0)	7 (28.0)	3 (12.0)	1 (4.0)	0	0	0
Grogginess after the procedure	18 (72.0)	4 (16.0)	2 (8.0)	1 (4.0)	0	0	0
How tired you felt after the procedure	12 (48.0)	6 (24.0)	4 (16.0)	3 (12.0)	0	0	0
Your ability to think clearly after the procedure	17 (68.0)	7 (28.0)	0	0	1 (4.0)	0	0
Ease of recovery after the procedure	23 (92.0)	0	1 (4.0)	1 (4.0)	0	0	0
How fast you returned to your usual daily activities (things you do everyday)	17 (70.8)	3 (12.5)	2 (8.3)	0	2 (8.3)	0	0
Your overall satisfaction with the sedation experience	24 (96.0)	1 (4.0)	0	0	0	0	0

Percentages based on the number of subjects that responded.

## Data Availability

The data presented in this study are available on request from the corresponding author. The data are not publicly available due to privacy restrictions.
